# Women’s Status and its Association With Home Delivery: A Cross-Sectional Study Conducted in Khyber-Pakhtunkhwa, Pakistan

**DOI:** 10.1007/s10995-021-03294-1

**Published:** 2022-01-04

**Authors:** Hussain Ali, Qaisar Khalid Mahmood, Aisha Jalil, Florian Fischer

**Affiliations:** 1 Abdul Wali Khan University, Mardan, Pakistan; 2 International Islamic University, Islamabad, Pakistan; 3 University of Lahore, Lahore, Pakistan; 4 Charité – Universitätsmedizin Berlin, Berlin, Germany; 5 Ravensburg-Weingarten University of Applied Sciences, Weingarten, Germany

**Keywords:** Pregnancy, Childbirth, Maternal health, Empowerment, Developing country

## Abstract

**Introduction:**

Home delivery is a predominant driver of maternal and neonatal deaths in developing countries. Despite the efforts of international organizations in Pakistan, home childbirth is common in the remote and rural areas of Khyber Pakhtunkhwa province. We studied women’s position within the household (socio-economic dependence, maternal health decision making, and social mobility) and its association with the preference for home delivery.

**Methods:**

We conducted a cross-sectional household survey among 503 ever-married women of reproductive age (15–49 years), who have had childbirth in the last twelve months or were pregnant (more than 6 months) at the time of the interview. A two-stage cluster sampling technique has been used for recruitment. Descriptive and bivariate analyses have been conducted. A binary logistic regression model was calculated to present odds ratios and corresponding 95% confidence intervals for factor associated with home delivery.

**Results:**

An inferior status of women, restrictions in mobility and limited power in decision making related to household purchases, maternal health care, and outdoor socializing are contributing factors of home delivery. Furthermore, women having faced intimate partner violence were much more likely to deliver at home (OR = 2.66, 95% CI: 1.83.3.86, p < 0.001).

**Discussion:**

We concluded that women are in a position with minimal authority in decision making to access and deliver the baby in any health facility. We recommend that the government should ensure the availability of health facilities in nearby locations to increase institutional deliveries in the study area.

## Significance

This manuscript is based on primary data collected in Pakistan. It deals with factors associated with the place of delivery. In Pakistan, there is a high rate of home deliveries which pose a challenge and risk to mothers and newborns. For that reason, it is important to provide evidence in this area for future public health actions.

## Introduction

Around 53 million deliveries take place without professional health service providers every year (WHO, 2012). Almost 50% of these deliveries are attended by traditional birth attendants (TBA) in developing countries. Globally, about 400 deaths occur per 100,000 live births annually (Shah & Say, 2007; WHO et al., 2007), with 99% in developing countries (Nour, 2008). Nearly one third of these deaths occur due to postpartum hemorrhage few hours after delivery, and about 50% within the first twenty-four hours of postpartum (WHO, 2005). Empirical evidence showed that the deaths are associated with the complications in prenatal, delivery, and postpartum period which cannot be handled by TBAs (Prata et al., 2011). Pakistan is one of the countries with the highest rate of maternal mortality (Ali, 2020; Kassebaum et al., 2016).

Previous research demonstrated the strong preference among women in developing countries, especially in rural regions, for home delivery (Hassan et al., 2012). According to Pakistan Demographic and Health Survey, more than 50% of deliveries take place at home (National Institute of Population Studies and ICF International 2013). Likewise, in Thailand, roughly 50% of deliveries are attended by TBAs at home, despite the availability of health facilities and services in the local community with very low financial costs (Ishikawaa et al., 2002). Similar sociocultural patterns are observable in our study area, the Khyber Pakhtunkhwa province, where 41% of pregnant women are assisted by TBAs and 6% by female relatives in their homes (National Institute of Population Studies and ICF International 2013).

Several studies conducted in *Sindh,* a province of Pakistan, highlighted that TBAs are performing unsafe and harmful delivery procedures (Ali, 2020; Fatmi et al., 2005; Fikree & Pasha, 2004). Besides the three core factors of poor maternal and child health, such as malnutrition, early marriage and fertility rate that contribute to poor maternal health outcomes in developing countries, is home childbirth. The practice of home childbirth is closely linked with misconceptions and lack of understanding of safe medical care procedures (Bhutta & Hafeez, 2015). Women are confined to the domestic sphere for delivery due to their low status and limited decision making.

Utilization of maternal health care services is one target set in Sustainable Development Goals and Millennium Development Goals to reduce maternal mortality. Many research studies revealed that women’s mobility during pregnancy is shameful for male family members. Therefore, they are restricted to “four-wall for delivery” [Literal meaning of a phrase used in local language] which are attended by TBAs. Women are even endangered of facing intimate partner violence in case of demand for hospitalization and other maternal health care services (Ali, 2020; Mumtaz & Salway, 2007).

A plethora of empirical evidence has confirmed that gender inequities affect maternal access to health care services, especially safe delivery procedures (Ali et al., 2016; Anna et al., 2016). The studies in developing countries like Nepal (Arati & Vijayan, 2017) and Uganda (Sarah, 2016) highlighted that the socio-cultural factors determine maternal and child health. However, evidence is not available from Khyber Pakhtunkhwa province of Pakistan. Other research shows that women at home have no say about their maternal health care decisions and need the approval of male family members (Ali et al., 2018; Shaikh et al., 2008). In the United States of America, a study was conducted near the Mexican border that demonstrated similar trends of childbirth decision making in association with women’s knowledge about birth and participation in decision making (Ali et al., 2018; Carla et al., 2016).

There have been lots of debates on the male dominancy that compels women to obey the decisions taken by their male family members (Ahmed et al., 2000). Several studies found that the possibility of exposure to and medical checkup by male health service providers is one of the major social factors which determine the home delivery preference (AbouZahr, 2003; UN, 2008). Furthermore, early marriage leads to discontinuation of education attainment among minor girls (Ali et al., 2016; Winnie et al., 2017).

This study analyzes the association between women’s social position and preference to home delivery. We contribute to the existing body of knowledge on public health policy and poor childbirth conditions prevailing in the less developed regions.

## Theoretical Framework

The health care utilization choice is one of the challenging determinants. Many factors are influencing the choice of maternal health care utilization. The influencing factors are including both sociocultural aspects and accessibility. Many researchers developed various models and theories to understand maternal health care utilization. The theories include illness and medical case theory introduced by Suchman (1965) and the self-help seeking theory developed by Mechanic (1978). Besides these theories, various models are also introduced to understand the health care utilization and accessibility. The choice making model by Young and Young-Garro (1982), the health behavior model by Andersen (1995) and the Three-delay model by Thaddeus and Maine (1994) were used.

**Fig. 1:**
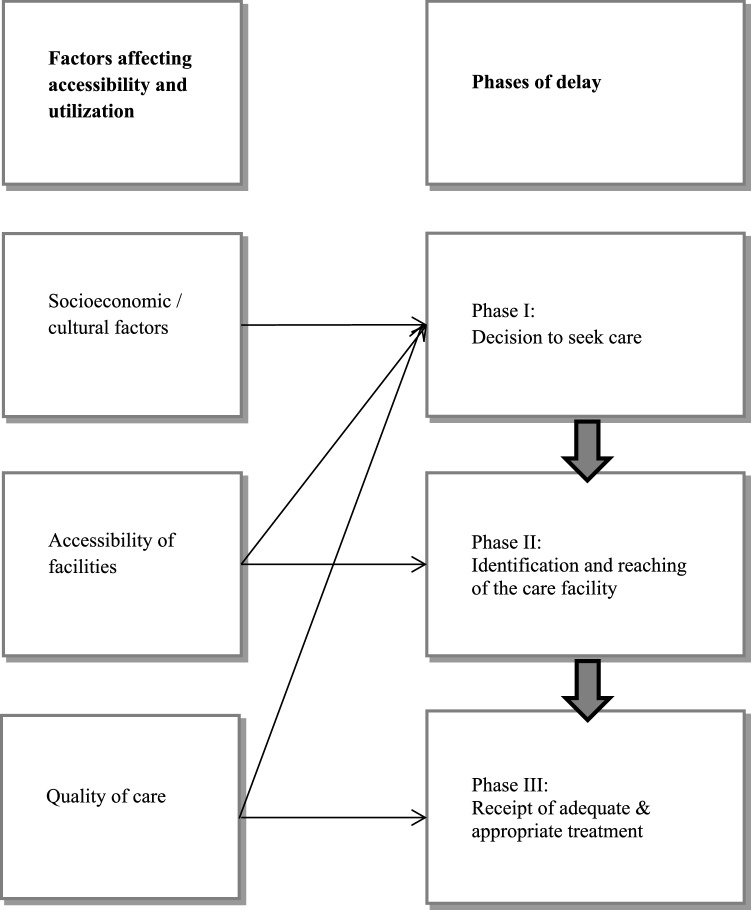
Three-delay model (3D’s Model) by Thaddeus and Maine (1994)

We applied the Three-delay model, which is based on three phases of delay. The first phase covers the women delay in decision to seek care. The delay is influenced by various factors including women low status, no knowledge about complication as outcome of pregnancy, acceptance of home delivery, maternal mortality and financial poor situation of the family. The second phase of the three-delay model is delay in reaching the care facility. The second phase of delay occurs due to the factors of distance from health facility, transportation unavailability, poor infrastructure and most of the residence area is mountains. The third phase of the model covers the delay of receiving adequate health care. The responsible factors are poor medical facilities, untrained and non-serious health service providers. The referral mechanism and emergency management of pregnancy and delivery complications are missing in the province (Fig. 1).

## Methods

### Study Design

We adopted a cross-sectional survey design in the study. The district health department shared the statistics from available records that 5000 married women in the age of 15–49 years have been pregnant in the district at the time the study was conducted. We selected 10% of the study population as a sample population in the study. Data from 503 ever-married women aged 15–49 years, who have had childbirth in the last twelve months or were pregnant (more than 6 months) at the time of the interview, living in households in rural and urban areas in Malakand, Khyber Pakhtunkhwa, Pakistan, were collected. The sample was drawn from available statistical records maintained in the District Headquarter Hospital, Tehsil Headquarter Hospital, Basic Health Units and records on TBAs in the study area. The Provincial Health Directorate appointed Lady Health Workers (LHWs) in all the districts of the province. The LHWs are visiting married women in every household in their duty locality to record the current status of pregnancy, antenatal care visits, place of delivery, postpartum checkup, complications faced by married women during pregnancy, delivery, and postpartum period. The LHWs submit the written statistical record every month to the Lady Supervisor in Tehsil Headquarter Hospital. The complete record is compiled in District Headquarter Hospital.

The data was collected through interviews, because most of the women were illiterate or had low levels of education. In the study area, due to strict cultural restrictions, interaction between men and women is not permissible. Therefore, we hired the services of six LHWs as research assistants to collect data from married women. The LHWs were selected in consultation with the district health department. We conducted an orientation session with hired LWHs and trained them about the data collection process. During this session they were educated on the purpose and objectives of the study. The LHWs were selected for household surveys because they had a good rapport with women in the research area. Furthermore, those LHWs were selected who permanently belonged to the research community because the respondents felt more comfortable with these LHWs as compared to women from outside the research community. Only female health workers were chosen to avoid charges of bias of responses due to the gender of the interviewer.

Both oral and written consent was obtained from each respondent prior to the interview. We informed the respondents about the study objectives and significance. We provided a free choice to all the respondents either to participate or not.

### Sampling

**Fig. 2:**
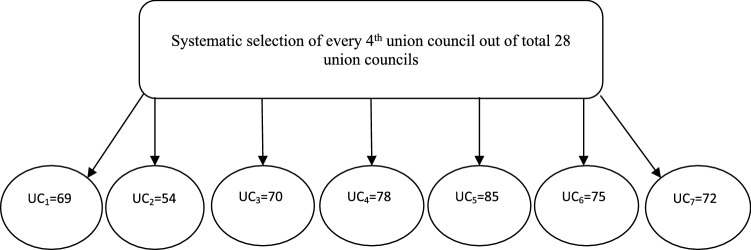
Sampling strategy

We used a two-stage cluster sampling technique to select the sample. In first stage, the study area was divided into urban and rural localities/union councils. In the second stage, women were selected for interviews. The localities comprised of 28 total union councils (administrative units). We used systematic sampling technique: Every fourth union council is selected from an official list of the district government department. We systematically selected 7 union councils out of a total 28 union councils. The selected union councils covered all geographical locations in the study area. We developed a data collection plan for the hired research assistants (LHWs) and a group of two LHWs each were made to collect household survey data from the sample in systematically selected administrative units. LHWs randomly visited the household and they interviewed currently married women at the age of 15–49 years with current pregnancy or delivery in last twelve months. The sample was proportionally divided in the selected seven union councils (Fig. 2).

### Dependent Variable

The place of childbirth for the last child born is the dependent variable in this study. The childbirth in hospital is considered as an adequate place of delivery and the childbirth at home as an inadequate place of delivery.

### Independent Variables

*Status of women*: We asked the respondents about their status as compared to their husbands. They were responded on two response categories as either inferior or equal to men in *Pakhtun* society.

*Women’s mobility*: Mobility refers to women’s ability to go outside the home independently. We categorized women mobility into two categories, being restricted to go outside and can go outside with husband or male companion. In other words, their mobility is fully restricted and they have less mobility in comparison with male members of the household. In traditional Muslim societies, women are not allowed to go outside the home because of purdah. Most of the times, those women who observe purdah rely on male members of the family when they want to go outside. We asked the respondents about purdah restriction in the family when they went outside.

Reasons of early age marriage: Early age marriage is one of the major cultural factors of poor maternal health care. In this study, we opted two reasons of early age marriage: 1) women’s position in the society and 2) women have no right to choose their spouses in Pakhtun society.

*Intimate partner violence*: Intimate partner violence is a major cause of poor maternal health care utilization. Women prefer to give birth at home in order to avoid intimate partner violence. We asked the respondents if they faced intimate partner violence during pregnancy.

*Decision making authority*: We measured the women’s decision making authority in household purchase, access to maternal health care services and outdoor socializing.

### Data Analysis

We started data entry parallel with household data collection on daily basis. When during data entry missing information was found, we tried to solve this issue with research assistants to collect or clarify about the missing information. Once the household survey was finished, we completed data entry in Statistical Package of Social Sciences (SPSS). We cleaned the entered data and then properly coded all the entered questions. Furthermore, we categorized and re-coded some open-ended questions for statistical analysis.

We applied descriptive analysis and bivariate logistic regression modelling to assess the association of variables. In descriptive statistics, the socio-demographic characteristics of currently married women were determined. We used bivariate analysis to found statistical relationships between the dependent variable and independent variables. We used odds ratios (OR) and 95% confidence intervals (CI) with a level of significance at 0.05.

## Results

### Descriptive Analysis

Overall, the childbirths have almost been equally distributed among home (n = 268; 53.3%) and hospital facilities (n = 235; 46.7%). Among the 503 respondents, around 43% of the women were 15–30 years. Almost all women were currently married and 90.3% were living with their husband. More than one-third (40.0%) married at the age of 14–18 years, 45.5% in the age of 19–23 years, and only 2.0% after 28 years of age. More than fifty percent (53.7%) of women had no or primary education. Similarly, among husbands, 31.0% had no or primary education, 41.7% had passed secondary education, and more than one-fourth (27.2%) passed intermediate. The majority (81.9%) was not employed in paid work and only engaged in routine domestic unpaid work. Most of the respondents had a poor socio-economic status. Nearly one-fourth (24.3%) had an income between 40,001 and 50,000 Pakistani Rupees, while only 9.7% had an income of more than 50,000 Pakistani Rupees. Our study also indicated that the majority (87.6%) were residing in joint family systems (Table 1).

**Table 1:** Socio-demographic characteristics of ever-married women aged 15–49 years

Characteristics	n	%
*Age of women (in years)*		
15–30	215	(43.1)
> 30	288	(56.9)
*Marital status*		
Married	498	(99.0)
Widowed	2	(0.4)
Divorced	3	(0.6)
*Living together with husband*		
Yes	454	(90.3)
No	49	(9.7)
*Age at marriage (in years)*		
14–18	201	(40.0)
19–23	229	(45.5)
24–28	63	(12.5)
> 28	10	(2.0)
*Women’s education*		
None or primary	270	(53.7)
Secondary	157	(31.2)
Intermediate and above	76	(15.1)
*Husband’s education*		
None or primary	156	(31.0)
Secondary	210	(41.7)
Intermediate and above	137	(27.2)
*Area of residence*		
Plain	406	(80.7)
Mountains	49	(9.7)
Semi-plain	48	(9.5)
*Women’s occupation*		
Employed	91	(18.1)
Housewife	412	(81.9)
*Monthly income (in Pakistani Rupees)*		
1–10,000	64	(12.7)
10,001–20,000	83	(16.5)
20,001–30,000	119	(23.7)
30,001–40,000	65	(12.9)
40,001–50,000	122	(24.3)
> 50,000	49	(9.7)
*Type of family*		
Nuclear	47	(9.3)
Joint	441	(87.6)
Extended	15	(2.9)
*Women’s tongue*		
Pushto	493	(98.0)
Other than Pushto	10	(2.0)

### Bivariate Logistic Regression

Table 2 shows both bivariate results based on cross-tables and the logistic regression model for home delivery as outcome variable and its associated factors. Women with an inferior status compared to men had a 2.33 timer higher likelihood (95% CI: 1.55–3.51, p < 0.001) for home delivery. Furthermore, a statistically significant difference was found for restrictions in mobility (OR = 1.87, 95% CI: 1.16–3.02, p = 0.010) and early marriage preference. In the selected population, a statistically significant difference was found for the place of delivery among women with their position in family as the main reason for early marriage compared to women who have had an institutional delivery in a hospital. The odds ratio for women with *Purdha* restriction was statistically not significant, indicating no difference between home and hospital delivery (p = 0.251). Women having faced intimate partner violence were much more likely to deliver at home (OR = 2.66, 95% CI: 1.83–3.86, p < 0.001).

**Table 2:** Factors associated with home delivery

	Place of delivery			
	Hospital facility	Home	OR (95% CI)^1^	p-value
	n (%)	n (%)		
*Women’s status*				
Inferior than men	139 (64.1)	216 (80.6)	2.33 (1.55–3.51)	< 0.001
Equal or superior to men	78 (35.9)	52 (19.4)	1.00	
*Restrictions in mobility*				
Lesser than men	157 (83.1)	165 (72.4)	1.00	
Totally restricted	32 (16.9)	63 (27.6)	1.87 (1.16–3.02)	0.010
Marriage preference				
*Right to choose the spouse*	86 (41.3)	143 (55.4)	1.76 (1.22–2.55)	0.003
No right to choose the spouse	122 (58.7)	115 (44.6)	1.00	
*Purdha* restriction				
Yes	123 (58.9)	169 (64.0)	1.24 (0.86–1.81)	0.251
No	86 (41.1)	95 (36.0)	1.00	
*Intimate partner violence*				
Yes	90 (42.3)	175 (66.0)	2.66 (1.83–3.86)	< 0.001
No	123 (57.7)	90 (34.0)	1.00	
*Decision making in household purchases/affairs*				
Husband	94 (43.3)	167 (62.3)	2.16 (1.50–3.12)	< 0.001
Jointly	123 (56.7)	101 (37.7)	1.00	
*Decision making for maternal health care*				
Husband	131 (64.2)	198 (76.4)	1.81 (1.21–2.71)	0.004
Jointly	73 (35.8)	61 (23.6)	1.00	
*Decision making for outdoor socializing*				
Husband	140 (64.5)	238 (88.8)	4.36 (2.73–6.99)	< 0.001
Jointly	77 (35.5)	30 (11.2)	1.00	

^1^Reference group: Delivery at hospital facility

If the decision for household purchases (OR = 2.16, 95% CI: 1.50–3.12, p < 0.001), maternal health care (OR = 1.81, 95% CI: 1.21–2.71, p = 0.004) and outdoor socializing (OR = 4.36, 95% CI: 2.73–6.99, p < 0.001) is with the husband instead of making joint decisions, the likelihood is much higher for delivering at home.

## Discussion

Our study revealed that women’s position in the family including an overall inferior status of women, restrictions in mobility, early marriage preference, intimate partner violence, limited decision making for maternal health care, limited decision making in household purchases/affairs and decision to visits outdoor socialize are significantly associated with the place of delivery among *Pakhtun* women.

Women have been subject to structural factors that determine their public sphere mobility and choice of place of delivery. Women largely are lacking access to public health facilities at the time of childbirth in our study area. Other research studies conducted in Pakistan and similar contexts have highlighted that women restricted mobility contributes to home deliveries and exposure to the risks of delivery complications (Ahmed et al., 2000).

We found that women living in mountainous rural areas are more likely to deliver their child at home than at some health facility due to a lack of means of transportation. Furthermore, in developing countries women residing in rural mountains areas are frequently not involved in decision making about their access to maternal health care facilities in the area and they are compelled to be attended by TBAs (Jokhio et al., 2005).

Another study conducted in Malakand, Pakistan, also demonstrated that Pashtun women have a status in household affairs with no or limited decision making (Naz et al., 2011). Researchers have attributed this weak position with respect to restriction to domestic sphere, socioeconomic dependence and decision making, due to which they take delivery at home by unskilled birth attendants (Iyayi, 2009). Many scholars have confirmed that a lack of autonomy negatively affects the maternal health conditions (Bhutta, 2004).

In our study area, early marriage among girls is highly prevalent. Early marriages among women contributing to complications in pregnancy and delivery. Many research studies also demonstrated that early marriage preference and practice is one of the major contributors to poor maternal health care among pregnant women in Pakistan, India and Nepal (Nasrullah et al., 2014). Evidence shows that early marriage preference and practice still prevail which not only reduces women’s access to education and other basic services but also increases the maternal mortality (Perveen, 2009). In our study area, men are the key decision makers about the household purchase. In the developing countries including Pakistan most of the decisions regarding household purchase, access to maternal health care services and use of family planning methods are taken by men (Fatmi et al., 2005; Kadir et al., 2003). Likewise, Fatmiet al. (2005) stated that women are not permitted to access the public sphere without the consent of male family members in Pakistan.

The intimate partner violence was highlighted by many researchers and a strong association was found with poor maternal health outcomes and home delivery in rural societies (Cripe et al., 2008; Pallitto et al., 2013). In male dominant societies women are kept poor and they are more vulnerable to intimate violence and restricted from access and utilization of health services (Ali et al., 2011; Sen & Östlin, 2008).

## Limitations

This study has some limitations. The study results are only applicable to *Pakthun* communities and cannot be generalized to other communities. The status of women within families in Pakistan need further investigation, particularly qualitative studies for a better understanding of this issue. When interpreting our results, one needs to acknowledge that we have focused on women*s status within the family. However, further factors also impact on women’s decision to seek care or delivery in a facility (such as quality and accessibility of health care system), which have not been investigated in our study. Furthermore, the inferior status is a perception and, therefore, a subjective appraisal of women, which may lead to a bias. Overall, further longitudinal studies including a variety of factors are required to validate the results of this cross-sectional study.

## Conclusion

Most of the married women in the study area delivered their child at home. Furthermore, currently pregnant women mostly planned to deliver at home. Women in Pakistan are in a position with minimal decision making authority. In the study area early marriage among girls is a socially approved practice and their outer mobility is restricted. We recommend that the government should frame a law to legally restrict early marriages among girl. In addition, the government should ensure the availability of health facilities in nearby locations to increase institutional deliveries. Awareness and public service messages should be on-air for general masses awareness about the pregnancy complications and preference of institutional delivery. The results can be generalized in other communities in Pakistan and even in other countries with patriarchal structure. Further longitudinal studies are required to validate the results of this cross-sectional study.

## Data Availability

Data is available from authors upon reasonable request.

## Abbreviations

*CI*: Confidence interval
*KPK*: Khyber Pakhtunkhwa
*OR*: Odds ratio
*TBA*: Traditional birth attendant
